# Wiring up for controlled flight

**DOI:** 10.7554/eLife.95989

**Published:** 2024-03-04

**Authors:** Albert Cardona

**Affiliations:** 1 MRC Laboratory of Molecular Biology, University of Cambridge Cambridge United Kingdom; 2 Department of Physiology, Development and Neuroscience, University of Cambridge Cambridge United Kingdom

**Keywords:** visual system, optic flow, Lobula Plate Tangential cells, flight, neural circuits, *D. melanogaster*

## Abstract

A map showing how neurons that process motion are wired together in the visual system of fruit flies provides new insights into how animals navigate and remain stable when flying.

**Related research article** Zhao A, Nern A, Koskela S, Dreher M, Erginkaya M, Laughland CW, Ludwigh H, Thomson A, Hoeller J, Parekh R, Romani S, Bock DD, Chiappe E, Reiser MB. 2024. A comprehensive neuroanatomical survey of the *Drosophila* Lobula Plate Tangential Neurons with predictions for their optic flow sensitivity. *eLife* **13**:RP93659. doi: 10.7554/eLife.93659.

While birds, bats and insects make flying look easy, this skill is anything but. As animals launch themselves into flight, a world that was once static, slow and fairly predictable is now suddenly fast and roaring. Suspended in air, which itself is in motion and often turbulent, animals must rely on what they see around them to work out where they want to go, and stabilize their bodies relative to the ground and their surroundings.

To achieve this, animals rely on optic flow: this is the apparent motion of objects in their visual field as they travel through an environment, such as the landscape swooshing past a bird as it flies. Optic flow is key to successful flight, as it allows animals to maintain a steady altitude and to negotiate obstacles while rapidly zooming through complex landscapes, such as a forest full of branches. This is why insects often smash into glass windows because these, being transparent, do not trigger any optic flow.

How animals in flight perceive and use optic flow to adjust their own motion relative to their surroundings has fascinated scientists for a very long time. This process starts in the retina with photoreceptors that detect changes in light and relay this visual information – via a network of neurons – to a region of the brain known as the central complex, which directs where the animal will move based on where it is in space (Rivera-Alba et al., 2011; Heinze, 2021; Seelig and Jayaraman, 2015; Franconville et al., 2018; Hulse et al., 2021). One way to better understand how this circuit of neurons supports flight is to investigate how they are wired together in the brain of the fruit fly *Drosophila melanogaster*, which has been imaged to a nanometre resolution using electron microscopy (Zheng et al., 2018).

Early studies of the fruit fly optic lobe – where visual information is first processed – revealed a modular arrangement with repeated vertical cartridges of neurons, one for each unit (known as an omatidium) of the insect’s eye (Meinertzhagen and O’Neil, 1991; Nern et al., 2015). Each cartridge is analogous to a single camera pixel. But these cartridges are not passive, each one computes changes in light intensity over time and, together with inputs from adjacent cartridges, the direction of motion of objects at a specific point in the fly’s field of view (Behnia et al., 2014; Maisak et al., 2013; Haag et al., 2016; Hassenstein and Reichardt, 1956; Takemura et al., 2013). While neural circuits spanning across adjacent cartridges largely consist of modular repeats, there are differences that contribute to their visual processing properties (Cornean et al., 2024).

A class of neurons in the optic lobe called Lobula Plate Tangential cells (LTPs for short) have been shown to process visual information related to movement. LTPs receive inputs from multiple cartridges, allowing them to relay to the central brain a general picture of how objects are moving across the fly’s whole field of view. Most studies have focused on LTPs that process vertical or horizontal motion (which is needed, for example, to detect drift from wind gusts). However, there is also a larger population of LTPs that are sensitive to optic flow, which are less well defined due to them being more challenging to study. Now, in eLife, Michael Reiser and colleagues – including Arthur Zhao as first author – report the location and connections of all LTPs in the visual system to better understand how flies process optic flow (Zhao et al., 2024).

The team mapped the complete set of LTPs in the fly’s eye from electron microscopy data. They then combined this information with an existing map of all other neurons in the optic lobe to determine the input field and optic flow properties of each LTP. While some LTPs were already known to encode horizontal, vertical or rotational movement, this work means that now all LTPs in the fruit fly brain have a predicted function. Zhao et al. (who are based at the Janelia Research Campus, Champalimaud Centre for the Unknown, and University of Vermont) also determined which neurotransmitters each LTP released by directly analysing the anatomical features of synapses observed in electron microscopy images (Eckstein et al., 2020).

Zhao et al. followed the trajectory of the LTP axons as they entered the central brain and identified six target regions, which may each control different types of movement response. The interconnectivity among LTPs, and their convergence into one of the six brain regions, also provides further evidence that the brain can compose more complex optic flow fields than those sensed individually by each LTP.

While the fruit fly is an excellent laboratory animal for studying the brain and behaviour (Pfeiffer et al., 2010; Dorkenwald et al., 2023), much insight into the visual control of flight came from studies in the much larger blowfly. Indeed, electrophysiological recordings of individual LTPs in blowflies yielded early insights into the properties of visual circuits in insects (Borst and Egelhaaf, 1990). Intriguingly, Zhao et al. report having found 58 LTPs in each brain hemisphere, a number almost identical to the ~60 reported in the blowfly. Far from a coincidence, this similarity reveals a fundamental arrangement of neurons in the visual system across fly species. This suggests that although a larger retina may provide higher visual acuity, the central brain will still extract a similar quality of optic flow information across its visual field regardless of the dimensions of the retina. Whether there is a limit to how reduced the retina can be before optic flow information is compromised may soon be revealed by connectomic studies in miniature insects such as the fairy wasp *Megaphragma sp.* (Polilov, 2012).

Nevertheless, the comprehensive map of all LTPs in the fruit fly brain provided by Zhou et al., together with the neurotransmitter signature of each neuron and the complete set of neurons they connect with, sets a new, outstanding baseline for studying how the brain uses optic flow signals to drive behaviour.

## References

[bib1] Behnia R, Clark DA, Carter AG, Clandinin TR, Desplan C (2014). Processing properties of ON and OFF pathways for *Drosophila* motion detection. Nature.

[bib2] Borst A, Egelhaaf M (1990). Direction selectivity of blowfly motion-sensitive neurons is computed in a two-stage process. PNAS.

[bib3] Cornean J, Molina-Obando S, Gür B, Bast A, Ramos-Traslosheros G, Chojetzki J, Lörsch L, Ioannidou M, Taneja R, Schnaitmann C, Silies M (2024). Heterogeneity of synaptic connectivity in the fly visual system. Nature Communications.

[bib4] Dorkenwald S, Matsliah A, Sterling AR, Schlegel P, Yu SC, McKellar CE, Lin A, Costa M, Eichler K, Yin Y, Silversmith W, Schneider-Mizell C, Jordan CS, Brittain D, Halageri A, Kuehner K, Ogedengbe O, Morey R, Gager J, Kruk K, Perlman E, Yang R, Deutsch D, Bland D, Sorek M, Lu R, Macrina T, Lee K, Bae JA, Mu S, Nehoran B, Mitchell E, Popovych S, Wu J, Jia Z, Castro M, Kemnitz N, Ih D, Bates AS, Eckstein N, Funke J, Collman F, Bock DD, Jefferis G, Seung HS, Murthy M, FlyWire Consortium (2023). Neuronal wiring diagram of an adult brain. bioRxiv.

[bib5] Eckstein N, Bates AS, Champion A, Du M, Yin Y, Schlegel P, Lu AKY, Rymer T, Finley-May S, Paterson T, Parekh R, Dorkenwald S, Matsliah A, Yu SC, McKellar C, Sterling A, Eichler K, Costa M, Seung S, Murthy M, Hartenstein V, Jefferis G, Funke J (2020). Neurotransmitter classification from electron microscopy images at synaptic sites in *Drosophila melanogaster*. bioRxiv.

[bib6] Franconville R, Beron C, Jayaraman V (2018). Building a functional connectome of the *Drosophila* central complex. eLife.

[bib7] Haag J, Arenz A, Serbe E, Gabbiani F, Borst A (2016). Complementary mechanisms create direction selectivity in the fly. eLife.

[bib8] Hassenstein B, Reichardt W (1956). Systemtheoretische Analyse der Zeit-, Reihenfolgen- und Vorzeichenauswertung bei der Bewegungsperzeption des Rüsselkäfers Chlorophanus. Zeitschrift Für Naturforschung B.

[bib9] Heinze S (2021). Mapping the fly’s 'brain in the brain'. eLife.

[bib10] Hulse BK, Haberkern H, Franconville R, Turner-Evans D, Takemura SY, Wolff T, Noorman M, Dreher M, Dan C, Parekh R, Hermundstad AM, Rubin GM, Jayaraman V (2021). A connectome of the *Drosophila* central complex reveals network motifs suitable for flexible navigation and context-dependent action selection. eLife.

[bib11] Maisak MS, Haag J, Ammer G, Serbe E, Meier M, Leonhardt A, Schilling T, Bahl A, Rubin GM, Nern A, Dickson BJ, Reiff DF, Hopp E, Borst A (2013). A directional tuning map of *Drosophila* elementary motion detectors. Nature.

[bib12] Meinertzhagen IA, O’Neil SD (1991). Synaptic organization of columnar elements in the lamina of the wild type in *Drosophila melanogaster*. The Journal of Comparative Neurology.

[bib13] Nern A, Pfeiffer BD, Rubin GM (2015). Optimized tools for multicolor stochastic labeling reveal diverse stereotyped cell arrangements in the fly visual system. PNAS.

[bib14] Pfeiffer BD, Ngo TTB, Hibbard KL, Murphy C, Jenett A, Truman JW, Rubin GM (2010). Refinement of tools for targeted gene expression in *Drosophila*. Genetics.

[bib15] Polilov AA (2012). The smallest insects evolve anucleate neurons. Arthropod Structure & Development.

[bib16] Rivera-Alba M, Vitaladevuni SN, Mishchenko Y, Lu Z, Takemura S-Y, Scheffer L, Meinertzhagen IA, Chklovskii DB, de Polavieja GG (2011). Wiring economy and volume exclusion determine neuronal placement in the *Drosophila* brain. Current Biology.

[bib17] Seelig JD, Jayaraman V (2015). Neural dynamics for landmark orientation and angular path integration. Nature.

[bib18] Takemura S, Bharioke A, Lu Z, Nern A, Vitaladevuni S, Rivlin PK, Katz WT, Olbris DJ, Plaza SM, Winston P, Zhao T, Horne JA, Fetter RD, Takemura S, Blazek K, Chang L-A, Ogundeyi O, Saunders MA, Shapiro V, Sigmund C, Rubin GM, Scheffer LK, Meinertzhagen IA, Chklovskii DB (2013). A visual motion detection circuit suggested by *Drosophila* connectomics. Nature.

[bib19] Zhao A, Nern A, Koskela S, Dreher M, Erginkaya M, Laughland CW, Ludwigh H, Thomson A, Hoeller J, Parekh R, Romani S, Bock DD, Chiappe E, Reiser MB (2024). A comprehensive neuroanatomical survey of the *Drosophila* Lobula Plate Tangential Neurons with predictions for their optic flow sensitivity. eLife.

[bib20] Zheng Z, Lauritzen JS, Perlman E, Robinson CG, Nichols M, Milkie D, Torrens O, Price J, Fisher CB, Sharifi N, Calle-Schuler SA, Kmecova L, Ali IJ, Karsh B, Trautman ET, Bogovic JA, Hanslovsky P, Jefferis GSXE, Kazhdan M, Khairy K, Saalfeld S, Fetter RD, Bock DD (2018). A complete electron microscopy volume of the brain of adult *Drosophila melanogaster*. Cell.

